# The use of self-quantification systems for personal health information: big data management activities and prospects

**DOI:** 10.1186/2047-2501-3-S1-S1

**Published:** 2015-02-24

**Authors:** Manal Almalki, Kathleen Gray, Fernando Martin Sanchez

**Affiliations:** 1 Health and Biomedical Informatics Centre, University of Melbourne, Melbourne, 3010, Australia

**Keywords:** Self-quantification, self-tracking, self-monitoring, quantified self, quantification activities, big data, reflection, knowledge, self-activation, personal health informatics

## Abstract

**Background:**

Self-quantification is seen as an emerging paradigm for health care self-management. Self-quantification systems (SQS) can be used for tracking, monitoring, and quantifying health aspects including mental, emotional, physical, and social aspects in order to gain self-knowledge. However, there has been a lack of a systematic approach for conceptualising and mapping the essential activities that are undertaken by individuals who are using SQS in order to improve health outcomes.

In this paper, we propose a new model of personal health information self-quantification systems (PHI-SQS). PHI-SQS model describes two types of activities that individuals go through during their journey of health self-managed practice, which are 'self-quantification' and 'self-activation'.

**Objectives:**

In this paper, we aimed to examine thoroughly the first type of activity in PHI-SQS which is 'self-quantification'. Our objectives were to review the data management processes currently supported in a representative set of self-quantification tools and ancillary applications, and provide a systematic approach for conceptualising and mapping these processes with the individuals' activities.

**Method:**

We reviewed and compared eleven self-quantification tools and applications (Zeo Sleep Manager, Fitbit, Actipressure, MoodPanda, iBGStar, Sensaris Senspod, 23andMe, uBiome, Digifit, BodyTrack, and Wikilife), that collect three key health data types (Environmental exposure, Physiological patterns, Genetic traits). We investigated the interaction taking place at different data flow stages between the individual user and the self-quantification technology used.

**Findings:**

We found that these eleven self-quantification tools and applications represent two major tool types (primary and secondary self-quantification systems). In each type, the individuals experience different processes and activities which are substantially influenced by the technologies' data management capabilities.

**Conclusions:**

Self-quantification in personal health maintenance appears promising and exciting. However, more studies are needed to support its use in this field. The proposed model will in the future lead to developing a measure for assessing the effectiveness of interventions to support using SQS for health self-management (e.g., assessing the complexity of self-quantification activities, and activation of the individuals).

## Background

Self-quantification of personal health and wellness data may contribute to approaches to more self-managed health care [1]. In recent years the general public has become more health-conscious, due in part to network and wearable sensor technologies that enable the non-expert to easily capture and share significant health-related information on a daily basis as part of attempts at self-knowledge. Such activities have been described as self-tracking, self-quantification, self-monitoring, self-regulation, self-diagnosis, self-experiment, self-feedback, and self-improvement [1,2]. New functionalities in wearable devices and the associated apps enable individuals to measure vital signs, access analytical tools, and quantify data about themselves faster and more ubiquitously than ever before [3].

As these technologies have become more widely available, they have given rise to a worldwide social movement called the 'Quantified Self'. This movement substantially has embodied the idea of self-tracking and self-quantification through setting up groups, regular meetings, and conferences around the world [4]. As of May 2014, there are 162 quantified-self groups and 30,229 members in 117 cities and 38 countries around the world [5]. The first Quantified-Self group setup outside USA was in Sydney Australia on Feb 2010 [5]. The Quantified Self is defined as a movement to incorporate technology into data acquisition on aspects of a person's daily life in terms of inputs (e.g., food consumed, quality of surrounding air), states (e.g., mood, arousal, blood oxygen levels), and performance (mental and physical) [6].

The three common terms that are associated with the Quantified Self movement are 'self-tracking', 'self-quantification', and 'self-monitoring'. There is confusion about the definitions of these three terms as they are closely interrelated. We propose some distinctions. 'Self-tracking' refers to a plan for continuous or periodic data acquisition. Once these acquired data are converted into quantitative or qualitative representations, this is called 'self-quantification'. For example, keeping diaries of food intake is a form of self-tracking while and converting these collected data into numbers and categories is self-quantification. 'Self-monitoring', on the other hand, usually refers to watching a specific health factor to ensure keeping it at a satisfactory level as part of a medically supervised care plan, such in case of a diabetic person who is using a blood glucose meter to monitor the level of blood glucose. To sum up these definitions, we can say that self-quantification includes self-tracking but self-tracking does not necessarily become self-quantification. Self-quantification becomes self-monitoring when the practice is a recognised part of clinical care.

Individuals can be described 'self-trackers' and 'self-quantifiers' as they are undertaking tracking and quantification activities, or 'users' or 'consumers' as they are using quantified-self technologies. Synonyms that are used include life-hackers, lifeloggers, lifebloggers, or lifegloggers. We prefer not to describe them as 'patients' since most of them are not doing these activities under medical supervision.

The individual is typically using multiple self-quantification technologies, and generating and aggregating different data types (e.g., physiological, environmental and genetic data) over a long period of time [7]. Therefore, an individual's self-tracking practice can generate data that are big in themselves [8] in terms of volume, variety, and velocity [9], as follows:

• Volume - self-trackers can collect data of enormous amount of detail about health and wellbeing over a lifetime.

• Variety - self-trackers are interested in tracking a broad range of data types to discern patterns of health and wellbeing. Such data could be in the form of numbers generated by wearable sensors, nominal or categorical, ordinal scales, photos, notes, etc.

• Velocity - data may be processed and streamed to provide feedback and trigger services in critical time frames.

However, using self-quantification data to achieve useful health outcomes poses major challenges in terms of managing data and reflecting on data. Despite the attention attracted by the Quantified Self movement, the potential of self-quantification tools and applications to inform and improve health self-management remains largely untapped. One explanation is that for an individual to do it successfully involves the systematic understanding of the tools' or app's actions plus the complex undertaking of user activities. Thus, we propose a model that can help us to understand more clearly what is needed for individuals to integrate informed self-tracking into effective self-management of their health.

## Personal Health Information Self-Quantification System (PHI-SQS) model

**Figure 1:**
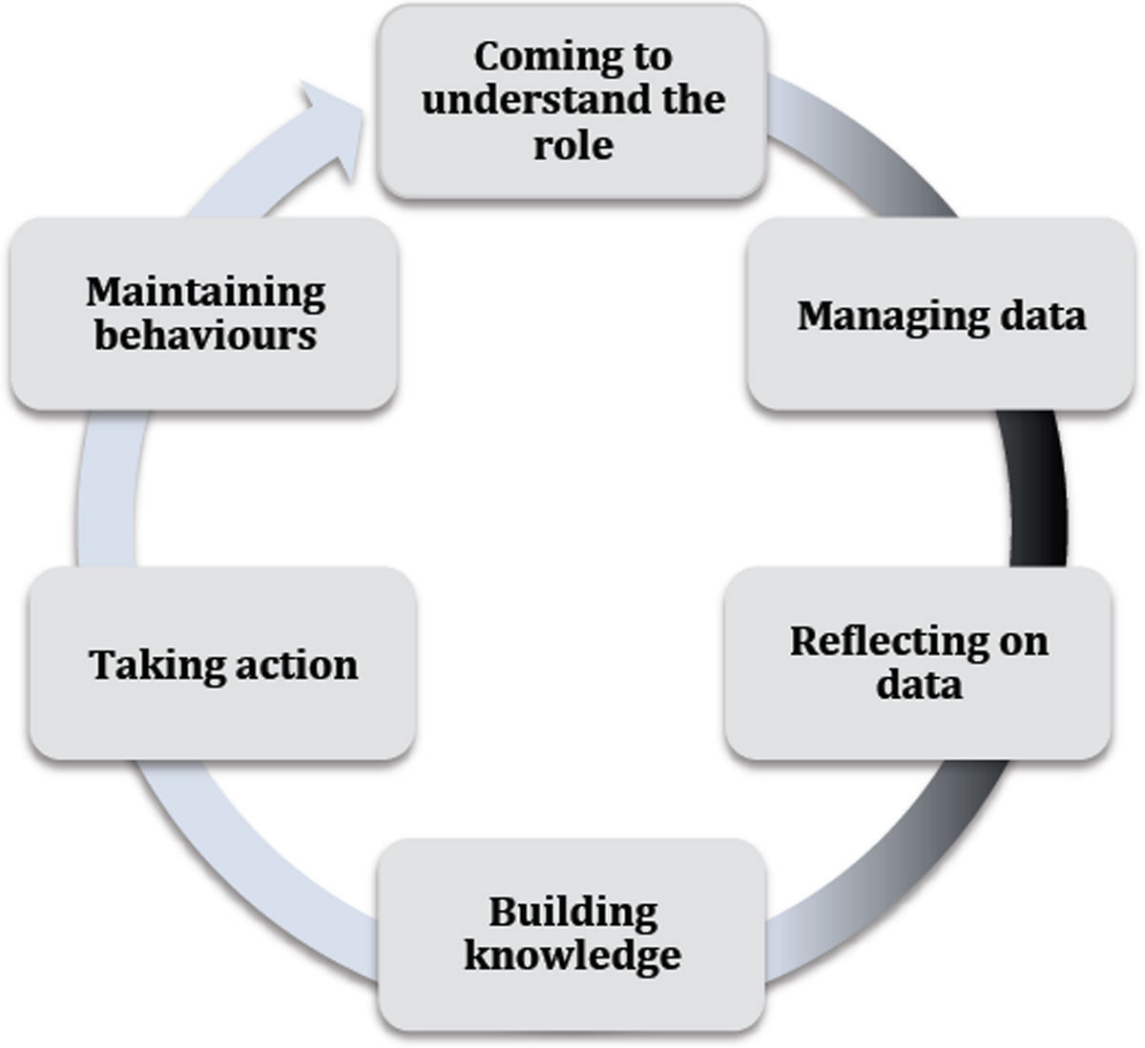
**PHI-SQS model**. Self-quantification activities are located in the dark side of the arrow, whereas self-activation activities are located in the light side.

The personal health information self-quantification system (PHI-SQS) model is proposed to illustrate essential activities that individuals go through during the journey of health self-management. PHI-SQS defines 'health self-management' as an activity in which the individual's objects (e.g., set of questions) are transferred into health outcomes through a series of processes, each process consisting of several stages and actions. These processes can be classified into two categories of activities: self-quantification and self-activation, as shown in Figure 1.

### Self-quantification activity

In this model, the self-quantification activity refers to the concept of creating consciousness and gaining self-awareness about one's health through having three key components: subject or individual self-tracker, the technology, and objectives in form of measurements or data. The main driver of the self-quantification activity is the transformation of the individual's object into measured units via the technology used. For example, wanting the answer to the question 'how much would I walk if I did not ride my bike?' makes the individual undertake certain activities such as collecting data and analysing them in order to generate the required measurements.

There are two essential stages that individuals undertake in this activity: Managing data, and reflecting upon the generated data and measurements. These stages involve sub-activities such as data collection, storing, organising, analysis, aggregation and sharing.

### Self-activation activity

In this model, the self-activation activity refers to the concept of taking a more active role in managing one's health through having three key components: beliefs, knowledge, and, skills [10]. The main driver of the self-activation activity is the transformation of the individual's gained knowledge into informed health outcomes.

There are four essential stages that the individuals go through in this activity: Coming to understand the role, building knowledge and confidence that are necessary to take action, knowledge translation into action, and maintaining behaviours.

The aim of this paper is to substantially and thoroughly discuss the stages of 'self-quantification activity' within the PHI-SQS model. Elaboration on the 'self-activation activity' stage is not included in this paper, but is the subject of a separate study that is still underway.

## Methods

We identified a representative sample of tools that were available in the Australian market in 2012. We selected eleven self-tracking tools and applications based on three categories of health data types. These tools and applications are: Zeo Sleep Manager [11], Fitbit [12], Actipressure [13], MoodPanda [14], iBGStar [15], Sensaris Senspod [16], 23andMe [17], uBiome [18], Digifit [19], BodyTrack [20], and Wikilife [21].

We classified these tools by health data types into exposome, phenome, and genome (Table 1): exposome refers to the lifelong exposure of an individual to environmental risk factors; phenome refers to the expression of a person's characteristics and traits as these are determined by the interplay of genetics and the environment; and genome refers to the hereditary instructions of a life form that are encoded in the DNA in the human being [22].

**Table 1:** Selected self-quantification systems by health data type.

	Environmental exposure (exposome)	Physiological patterns (phenome)	Genetic traits (genome)
**Self-Quantification Systems**	Zeo Fitbit Sensaris Senspod	Moodpanda Actipressure iBGStar	23andMe uBiome
	Digifit, Wikilife and BodyTrack		

We also classified the selected self-quantification systems into two groups based on their major distinguishing characteristics. We then discuss the data flow stages and user system interaction in these two types of SQS.

## Main findings

**Figure 2:**
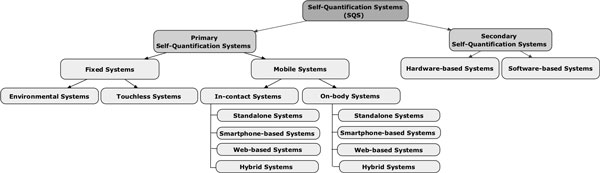
**SQS taxonomy**.

Self-quantification systems (SQS) can be classified into primary and secondary SQS (Figure 2). Each of these categories is further classified into sub-categories based on major distinguishing characteristics of these various systems which are: directness of capturing data from the user (Primary or Secondary systems), location of the sensors (Mobile or Fixed systems), invasiveness in taking measurements (In-contact or On-body systems), data type (Environmental or Touchless systems), location of data aggregation (Software-based or Hardware-based systems), location of data analysis and visualisation (Standalone, Hybrid, and Smartphone systems).

### SQS taxonomy

#### Primary SQS

A primary self-quantification system is a system that collects date directly from the users. It typically consists of a data-collecting unit (e.g., embedded sensors or a tangible device) for collecting data, and a data-processing unit (e.g., device, smartphone, computer, server, etc.) which runs the associated application (e.g., a smartphone-based app, computer-based software, web-based application, etc.) for analysing, visualising, and recording the history of the data, and/or sharing the collected data and findings. Zeo Sleep Manager, Fitbit Ultra Wireless Activity Tracker, FitLinxx Actipressure, MoodPanda, iBGStar, Sensaris Senspod, 23andMe, and uBiome are examples of primary self-quantification systems.

#### Taxonomy of primary SQS

The primary self-quantification systems can be classified into two groups, Fixed and Mobile self-quantification systems, based on the location aspect of the sensors.

In mobile self-quantification, the sensor is collecting data while it is installed or attached to a moving object such as a person (e.g., wearable sensors), bus, car, etc. However, in fixed self-quantification, the sensor is collecting data while it is installed or attached to a fixed place such as an office, home, clinic, etc.

#### Mobile SQS

Mobile self-quantification systems are further classified into two groups: Invasive sensors (in-contact sensors) and Non-invasive sensors (on-body sensors). This classification is based on whether the measurements involve inserting a tool under the skin or into a body cavity. The invasive sensors have tools for skin pricking by the patients, for example, taking a blood sample for testing the glucose level [23]. However, non-invasive sensors do not need the users to puncture the skin to take measurements.

#### Standalone, Hybrid, Smartphone, and Web-based SQS

Moreover, both of the invasive and non-invasive systems can be further classified into: Standalone systems, Hybrid systems, Smartphone-based systems, or Web-based systems. This classification is based on the location of data collection, analysis and visualisation, i.e. where such actions are taking place.

#### Standalone SQS

A standalone system can be described as an integrated data-processing unit which is a device that usually has a small screen, software, and embedded sensors. Data collection, analysis, and visualisation are taking place on the device. Such a system has the ability to capture data through the embedded sensors, analyse it locally and display the results on the device's screen.

In addition, it is not necessary to have an Internet connection or pair the device with other data-processing units such as computers or smartphones to see the captured measurements. However, to make use of these data, users can pair the device automatically/wirelessly or manually/wired with a data-processing unit - depending upon the device's connection specifications. Here the pairing up process is optional [24]. Garmin [25] and Suunto [26] are examples of the standalone systems. These devices can log distances in land, sea and air locations and measure performance for cyclists, hikers, divers and others.

#### Hybrid SQS

A hybrid system consists of a data-processing unit (e.g., smartphone, computer, device, etc.) which runs the associated application, and an external data-collecting unit (e.g., wristband, headband, etc.). The data-collecting unit collects and synchronises data in real-time with the data-processing unit where data analysis, visualisation, and other processing actions are undertaken. It is worth noting that in some systems such as in 23andMe and uBiome, data processing and analysis occur outside the application; and the application will be only for data visualising and sharing the findings.

What makes this type of systems different from the standalone systems is that the users have no way to see the collected data except through the associated application and data-processing unit. Thus, to make use of these data, the user has to start the application [24]. Adidas MiCoach [27] is an example of the hybrid systems. Adidas MiCoach X_CELL has a clip that can be worn on a waistband or chest strap. It captures the movement of the user during training but no measurements are displayed on the clip as it has no screen. The only way to see these measurements is through the paired smartphones and the associated app.

#### Smartphone-based SQS

A smartphone-based system consists of a data-processing unit which is the smartphone device, and an app that is run on the smartphone. Data collection, analysis, and visualisation take place on the smartphone. The smartphone device incorporates embedded sensors such as the Global Positioning System (GPS) and accelerators, and additional technology such as the camera and the keypad.

In this type of system, the app utilises the smartphone's capabilities for collecting data such as using the accelerators to sense the user's motions, and the keypad for entering data. Data are displayed on the smartphone screen through the app's interface [24]. In addition, this category includes tablet computers (e.g., iPad, Windows 7), and cellphones or PDAs (Personal digital assistant) (e.g., Nokia E90, LG Rumor Reflex).

iTreadMill [28], RunKeeper [29], and Endomondo [30] are examples of this systems type. These smartphone-based systems can track walking, running, cycling, and other physical activities.

#### Web-based SQS

A web-based system consists of a data-processing unit which is the remote server that runs the application/service; and a computing device (e.g., desktop, laptop, etc.) that has a Web browser for accessing the application over an internet connection.

Data collection is carried out by the user. S/he enters observational data via the device's keyboard into the application, whereas data processing and analysis are taking place on the server of the service provider. Data are displayed through the application's interface for interactive data-visualisation purposes. Chartmyself [31], TRAQS.me [32], and Statwing [33] are examples of such systems. These web-based systems can track almost anything via manual data logging. Also, some of these web applications have a mobile version for smartphones and tablets.

#### Fixed SQS

Fixed self-quantification systems can be divided into two groups: environmental and touchless systems based on the type of the captured data. Environmental systems record and measure environmental-related factors such as ambient temperature, precipitation rates, humidity, pollution, etc. Sensaris Senspod is an example of this type of systems.

On the other hand, touchless sensors take unobtrusive measurements of the user's activities and biomedical signals, for example, installing sensors in the user's bed for tracking ECG signals, weight, body movement, and snoring during sleep [34].

In such systems, a data-collecting unit is attached to a fixed place to collect data, and paired with a data-processing unit for analysing and displaying data. One potential benefit of using such systems is that the captured environmental-related data can be correlated with a user's health data to provide a comprehensive view of health status.

#### Examples of primary SQS

Table 2 shows the description and classification of the selected primary self-quantification systems.

**Table 2:** Examples of primary SQS and their classification.

Primary Self-Quantification System		
Components	Classification	Description
**Zeo Sleep Manager**		

Zeo headband sensor, Zeo bedside clock device, computer or smartphone, and Zeo application	Hybrid system	It tracks sleep quality; the amount of hours slept at each of the four different sleep stages (REM, deep sleep, light sleep, and waking).

**Fitbit**		

Fitbit clip, USB dongle, computer or smartphone, Fitbit application	Standalone system	It tracks movement, stairs climbed, showing the exact steps taken, calories burned, distance travelled, and hours of sleep.

**Actipressure**		

Inflatable rubber cuff, Actipressure device, FitLinxx SyncPoints and ActiHealth application	Hybrid system	It tracks blood pressure.

**MoodPanda**		

Smartphone and MoodPanda app	Smartphone-based system	It tracks the user happiness mood.

**iBGStar**		

Lancing tool, test strip, blood glucose meter, smartphone, and iBGStar® Diabetes Manager app	Hybrid system	It tracks the blood sugar.

**Sensaris Senspod**		

Sensor, computer or smartphone, and MobiSense application	Standalone system	It tracks Carbon monoxide (CO), Nitrogen oxide (NOx), Noise, Temperature, and Humidity.

**23andMe**		

Sample kit and 23andMe application	Hybrid system	It is a genetic test for DNA analysis.

**uBiome**		

Sample kit and uBiome application	Hybrid system	It provides an analysis of the microbes that exist in the skin, ears, mouth, sinuses, genitals and gut.

#### Secondary SQS

A secondary self-quantification system can be described as a tool - whether it is a tangible or intangible tool - for aggregating or integrating the collected data by a primary self-quantification system. Digifit, BodyTrack, and Wikilife are all examples of secondary self-quantification systems.

#### Taxonomy of secondary SQS

Secondary self-quantification systems are classified into two types: hardware-based systems, and software-based secondary systems. A hardware-based system consists of a device which is a connector for aggregating data that are captured by primary systems, and a web-based application for aggregating, visualising, and sharing data. In some cases, there is a mobile version of the web service (e.g., smartphone-based app). Digifit is an example of hardware-based systems.

On the other hand, a software-based system has mainly a web-based application for aggregating, visualising, and sharing tracked data. BodyTrack and Wikilife are examples of this type of tools.

#### Examples of secondary SQS

Table 3 shows the description of the secondary self-quantification systems examples.

**Table 3:** Examples of secondary SQS and their classification.

Secondary Self-Quantification System		
Components	Classification	Description
**Digifit**		

Digifit connector device, smartphone app, primary SQS, and website	Hardware-based system	Digifit is a cardio fitness system which consists of a connector that can aggregate heart rate and all runs, rides, spinning and cardio, and Digifit website. The connector device must be attached to a smartphone to start collecting data. Digifit is compatible with about 80 ANT+ sensors (ANT+ is an interoperability standard): Zeo, Fitbit, Garmin, Adidas, Withings and more.

**BodyTrack**		

BodyTrack website, primary SQS, and computer	Software-based system	BodyTrack website aggregates personal measurement readings - such as sleep quality, exercise, food intake, calories consumed, weight, environmental data etc. - that are delivered by different tools - such as Fitbit, and Zeo, etc.

**Wikilife**		

Wikilife website, primary SQS, and computer	Software-based system	Wikilife website aggregates lifestyle information such as exercise, health, psychological, nutrition, milestones (important events during an individual's lifetime), work, education, beauty, travel, spirituality, and physiological data.

### Discussion

In primary and secondary self-quantification systems, the individuals undertake different actions and activities. Considered altogether, these illustrate how complex and nuanced the practice of self-quantification is. Here we describe and discuss data flow stages and user system interactions in each of these different stages.

#### Data flow stages and activities in primary and secondary SQS

Data flow stages refer to the status of the data within the system under the effect of the User and System Interaction (USI). The data flows within the system in sequential stages from data collection, through data transmission, saving, storing, analysis, visualisation, to sharing data. Each of these stages would either represent an activity or remain just an action/process; this depends on whether there is a kind of USI occurring at this stage or not.

User and System Interaction (USI) refers to the kind of interaction between the system and its user such that the required activities (e.g., data collection, storing, etc.) would not be initiated or undertaken without the occurrence of this interaction. Such interaction is motivated by the user's objects and is mediated by the self-quantification tools and applications [35,36], and it generates data through transforming the user's objects into measurements.

Three properties can be used to differentiate the kind of interaction: user-driven action, system-driven action, and combination of both user and system actions [37]. A system-driven action is an action initiated and undertaken by the system. The user usually has limited interaction with the tools here. For example, in Zeo sleep manager, once the user puts the Zeo's headband on and turn the bedside-clock device on, the data are saved on the device's SD card over a Bluetooth network automatically by the system.

On the other hand, the user-driven action is an action initiated and undertaken manually by the user. The existence of the tool is not enough for performing the required action by the system. For example, in the MoodPanda app, the user needs to interact with the smartphone's keypad - the tool for collecting data - to manually enter data into the app.

The final type is the combination of both user and system actions. Here the intended action is initiated by the user and undertaken by the system or vice versa. For example, in Fitbit, once the user puts on the Fitbit clip to start collecting data, the data-collection action will be carried out automatically by the system. But also, in Fitbit, the system creates a record of data and allows the user to download it for storing purposes. Here the system initiates the record for the user, but the storing action is carried out by the user.

Activity refers to the purposeful action within USI that is initiated or undertaken by the user in order to accomplish a specific task [35,36].

In the sections that follow, we scrutinise each data flow stage and associated USI as follows:

• Describe the main action.

• Identify the tools that support the required action (i.e. wearable sensors and accelerometers are tools for performing the data-collection action).

• Identify the property of the interaction (system-driven action, user-driven action, or a combination of both).

• Identify activities. Unless the action is system-driven only, we can say that it represents an activity.

The aim of this analysis is to develop a systematic understanding of the main self-quantification activities that self-trackers undertake as these are influenced by the SQS data management capabilities. Tables (4 and 5) provide a summary of the data flow stages and activities in both primary and secondary SQS. Further details of this analysis can be found in [3].

**Table 4:** Data flow stages and activities in primary SQS.

Data flow stages			
Tool	SQS	Action property	Activity (Yes or No)
**Data collection**: action that generates data from and about the individual.			

Zeo headband sensor	Zeo sleep manager	C	Yes
Accelerometer and Altimeter sensor on Fitbit clip	Fitbit	C	Yes
Inflatable rubber cuff	Actipressure	U	Yes
IPhone keypad	MoodPanda	U	Yes
Blood glucose meter	iBGStar	C	Yes
Senspod device	Sensaris Senspod	C	Yes
Sample kit	23andMe and uBiome	U	Yes

**Data transmission**: action of transferring data from the data-collecting units to the data-processing units (e.g., smartphone) where they reside in a storage place temporarily or permanently.			

Bluetooth	Zeo sleep manager and Sensaris Senspod.	S	No
WiFi	Fitbit	C	Yes
BodyLAN wireless	Actipressure	S	No
Not applicable	MoodPanda	-	-
Universal Serial Bus (USB) network (e.g., blood glucose meter and its adaptor)	iBGStar	S	No
Shipping the sample kit	23andMe and uBiome.	U	Yes

**Data saving**: action of keeping data in a temporary storage place on the data-collecting units.			

Smartphone memory	MoodPana, and iBGstar.	S	No
Internal flash storage or SD Card	Zeo, Fitbit, Actipressure, and Sensaris Senspod.	S	No
Not applicable	23andMe and uBiome.	-	-

**Data storing**: action of keeping data in a long-term storage place for archiving purposes.			

Application has a feature for downloading data from the server of the service provider into files (e.g., XML, CSV, etc.)	Zeo, Fitbit, Actipressure, iBGstar, Sensaris Senspod, MoodPanda, 23andMe and uBiome.	C	Yes

**Data analysis**: action of converting the raw collected data into information via the data-processing unit's built-in algorithms.			

Device	Zeo, Fitbit, Actipressure, iBGstar, and Sensaris Senspod.	S	No
Smartphone-based app	MoodPanda	S	No

**Data visualisation**: action of transforming the collected and analysed data into graphical representations.			

Device display screen	Zeo, Fitbit, Actipressure, and iBGstar.	S	No
Smartphone-based app	Zeo, Fitbit, iBGstar, Sensaris Senspod, and MoodPanda.	C	Yes
Web-based application	Zeo, Fitbit, Actipressure, iBGstar, Sensaris Senspod, 23andMe and uBiome.	C	Yes

**Data sharing**: action of data exchanging and diffusing with either among different self-quantification systems, or with different people.			

Application has a feature for sharing data with social networks such as Facebook	Zeo, Fitbit, Actipressure, MoodPanda, iBGstar, and Sensaris Senspod.	C	Yes
Application has a feature for importing data; so the user can share them manually.	23andMe and uBiome.	U	Yes

S = System-driven action, U = User-driven action, C = Combination of both action

**Table 5:** Data flow stages and activities in secondary SQS.

Data flow stages			
Tool	SQS	Action property	Activity (Yes or No)
**Data collection**			

Primary self-quantification tools	Digifit, BodyTrack and Wikilife	C	Yes
Primary self-quantification tools and apps		U	Yes

**Data transmission**			

Universal Serial Bus (USB) network	Digifit, BodyTrack and Wikilife	S	No
Application has a feature for importing data		U	Yes

**Data storing**			

Files (e.g., XML, CSV, etc.)	Digifit website, BodyTrack and Wikilife	C	Yes

**Data aggregation**			

Connector	Digifit, BodyTrack and Wikilife	S	No
Web-based application		C	Yes

**Data visualisation**			

Web-based application	Digifit website, BodyTrack, and Wikilife	C	Yes

**Data sharing**			

Application has a feature for sharing data with social networks such as Facebook	Digifit website, BodyTrack, and Wikilife	C	Yes
Application has a feature for importing data		C	Yes

S = System-driven action, U = User-driven action, C = Combination of both action

### Data flow stages in primary SQS

#### Data collection

Data collection, the first stage in data flow, is the action that generates data from and about the individual; even this initial data acquisition action shows complexity. Table 4 shows the tools used in each different system as well as the different properties of the interaction with the user in this stage.

In Zeo, Fitbit, iBGStar, and Sensaris Senspod, the user initiates the action of data collection and the sensor performs the data-collection action. This kind of USI is of the combination of both user and system actions type. On the other hand, data collection in Actipressure, the MoodPanda app, 23andMe and uBiome requires a user-driven method, as the collection action is initiated and performed by the user aided by data-collecting tools such as an inflatable rubber cuff, the keyboard of a computer or smartphone, or a sample kit, respectively.

#### Data transmission

Data transmission is the action of transferring data from the data-collecting units to the data-processing units where they reside in a storage place temporarily or permanently.

Data transmission in Zeo, iBGStar, and Sensaris Senspod is mainly undertaken automatically by the system and therefore can be described as a system-driven action. In 23andMe and uBiome, the data transmission is user-driven - the user physically ships the sample kit which is the data-collecting unit to the 23andMe company and uBiome company in California for data analysis. However, in Actipressure and Fitbit, the data transmission action is a combination of user and system actions, where it is initiated by the user and performed by the system (Table 4). For keeping data in a temporary storage place we use the term data saving, whereas in a long-term storage place we use the term data storing, as described next.

#### Data saving

Data saving is the action of keeping data in a temporary storage place on the data-collecting units (e.g., wearable sensors) before they are offloaded to the paired data-processing units (e.g., smartphone, computer, server, etc.). This kind of action appears clearly in the hybrid type of SQS systems (Figure 2). In Zeo, Fitbit, iBGStar, Actipressure, Moodpanda, and Sensaris Senspod data are saved automatically (Table 4).

#### Data storing

Data storing, the action of keeping data in a long-term storage place, involves transferring data from the data-processing units to files on personal computer or a portable hard drive, or the Cloud, for example, where they reside for archiving purposes.

As self-tracking can happen over a long period of time, and as time passes and technology improves, people will probably move between services. Therefore, the users need to download their data and keep them on a secure single place. In this case, it is the user's responsibility to do storing activities such as organising, maintaining, keeping data up-to-date, etc.

In the eight primary self-quantification systems, the SQS system creates a record of the collected data, and the user then accesses these data and downloads them. Here the system starts the storing-action by initiating the record for the user, but the action of storing is carried out by the user. Thus, storing data is a combination of both user and system actions (Table 4).

#### Data analysis

Data analysis is the action of converting the collected raw data into information through utilising the data-processing unit's capabilities (e.g., built-in algorithms). Zeo, Fitbit, Actipressure, MoodPanda, iBGstar, and Sensaris Senspod have the ability to convert raw data into measurements. On the other hand, in 23andMe and uBiome, data analysis is performed by a group of researchers and experts, using single nucleotide polymorphisms (SNPs) technologies. Table 4 shows the data analysis classifications of these different SQS.

#### Data visualisation

Data visualisation is the action of transforming the collected and analysed data into graphical representations (e.g., trend charts, logbooks, statistics, infographics, etc.).

All eight primary self-quantification systems provide different kinds of data visualisation options. Zeo, Actipressure, MoodPanda, iBGstar, Sensaris Senspod, 23andMe, and uBiome have tools that allow the users to choose different viewing options (e.g., pie chart, scatter chart, table, line chart, area chart, etc.), and provide a history of the collected data as well. In this case, the user initiates the visualisation action by choosing a viewing option, and the system completes the action by rendering the visual artefact, Table 4.

#### Data sharing

Data sharing is the action of exchanging and diffusing data either among different self-quantification systems, or with different people. On one hand, sharing data with systems other than the one which generated the data is usually intended to aggregate multiple types of data in one place (e.g., blood glucose, physical activities, weather conditions, location, etc.) to build a complex representation, textual or graphical, of personal health information. On the other hand, sharing data with other people (e.g., with healthcare professionals, with family members, or with interest groups,) is often the basis for sharing experiences comparing performance and articulating lessons learned (Table 4).

### Data flow stages in secondary SQS

#### Data collection, transmission, and storing

Data collection action in secondary self-quantification systems shows increased complexity. In the hardware-based secondary systems (e.g., Digifit), the users must use a connector along with the primary SQS used (e.g., Fitbit) in order to enable the actions of data collection and aggregation. Next, the users must plug-in the connector to the processing-units (e.g., smartphone) to initiate the action of data transmission. The activity of collecting data in the hardware-based secondary SQS is classified as combination of both user and system actions

In the software-based secondary systems (e.g., BodyTrack and Wikilife), the activity of collecting data involves sub-activities such as exporting data from the primary SQS, storing them locally on a personal computer, and organising them in a way that makes accessing these data convenient. Next, the users must upload the data to a website in order to facilitate data aggregation and visualisation. The activity of collecting data in the software-based secondary SQS is classified as user-driven action (Table 5).

#### Data aggregation

Data aggregation is the action of combining the collected data into one place. In the Digifit data are aggregated by the connector, whereas in BodyTrack and Wikilife data are aggregated by the web-based application. The user activity in data aggregation involves uploading the collected datasets manually to these systems in order to facilitate a more comprehensive way of data visualisation. In Digifit, data-aggregation action is classified as a system-driven method. On the other hand, in BodyTrack and Wikilife, it is classified as a combination of both actions (Table 5).

#### Data visualisation and sharing

The description of these activities is the same as the description of the corresponding activities in the primary SQS. However, the capabilities of data visualisation in secondary SQS are more advanced than in the primary SQS as they enable the creation of more personalised dashboards and reports to track progress and explore multiple types of data. Table 5 shows the tools used by Digifit, BodyTrack, and Wikilife within each of these different stages.

## Systematising activities in the self-quantification stage of the PHI-SQS model

This discussion has served to show that the main activities in both primary and secondary SQS. Although the data flow stages are similar in different self-quantification systems, the individual users activities differ depending on the type of the SQS. In primary SQS activities are data collection; data transmission; data storing; data analysis and visualisation; and data sharing, whereas in secondary SQS the main activities are data collection; data storing; data aggregation; data visualisation; and data sharing.

Each of these activities has sub-activities and actions that an individual user must understand to use them effectively for health self-management, and these differ in their operation and complexity from one system to the other. Because our focus is on the individual user in this discussion, we have not gone into even further details about the activities and the associated challenges in both primary and secondary systems that a system designer would need to consider.

In summary, we can say that all of the illustrated activities in both primary and secondary self-quantification systems can be grouped into two categories: managing health-related data, and reflecting upon the cumulated measurements that indicate health status, as shown in Table 6.

**Table 6:** Self-quantification activities in PHI-SQS model.

Self-quantification activities	Data flow stages
**Individual (User system interaction)**	**Self-quantification system**

Managing data	Data collection
	Data transmission
	Data storing

Reflecting upon data	Data aggregation
	Data analysis and visualisation
	Data sharing

### Managing data as an activity in the model

This activity describes the processes and actions that are necessary to establish, use and maintain a mapping between the individual's needs and information [38]. In this type of activity, the individual is enabled to organise self-tracking data accumulated over a long period of time. By going through a series of sub-activities such as data collecting, transmission, storing, etc.

### Reflecting upon data as an activity in the model

Although managing data is complex in itself, nevertheless a person who is self-quantifying for health self-management needs to do even more than this with data. Reflection describes the processes and actions that are necessary to work with data to establish self-awareness of how aspects of one's daily life affect aspects of one's health. For example, looking at the blood glucose readings along with measurements of the level of physical activity may facilitate exploring the influence of these lifestyle factors on sleep quality, and then reflecting upon correlating these measurements with sleep measurements, perhaps even arriving at a new view of the interplay among all three health indicators. In this type of activity, the individual is empowered to find meaning in their self-tracking data through the use of tools for data aggregation, visualisation and sharing [39].

### Conclusion

Our SQS taxonomy, as shown in Figure 2, provides a hierarchical classification based on major distinguishing characteristics of a representative cross-section of self-quantification systems we examined. It helps to provide a deeper understanding of how individuals can expect to engage with the big data that are generated by the SQS they use within their self-quantification practice. Many new SQS products are coming onto the market, and we believe that the classification system we have developed will be applicable to all.

Our model of personal health information self-quantification system (PHI-SQS) relates the essential processes and activities that the individuals can expect to go through while using various self-quantification tools and apps on their health self-management journey.

In our research, this model fits within a larger framework for conceptualising all elements that belong to the health self-management environment (e.g., individuals, tools and applications, data and measurements, etc.); elaboration on the application of activity theory for this purpose is out of scope here but is the subject of a further paper. Also, further research is under way to test the real explanatory and predictive value of our PHI-SQS model among self-tracking individuals.

Finally, using self-quantification data to achieve useful health outcomes poses major challenges not only for the individual self-tracker. The individual can generate data that are big in themselves, and these massive data are amplified significantly if large numbers of people adopt self-tracking practice. Our further research aims to scope what is possible and test what is workable to improve health outcomes through the use of self-quantification systems not only the individual level but also the population level.

### Competing interests

The authors declare that they have no competing interests.

### Authors' contributions

MA reviewed the self-quantification tools and applications as well as developed the taxonomy of self-quantification systems and proposed the PHI-SQS model. MA also structured and wrote the manuscript. KG provided advice on conceptualisation and on the conclusion and other responses to reviewers. FMS reviewed the article and provided critical comments as well as advice on layout of figures and tables. All authors read and approved the final manuscript.

### Authors' information

Ms Manal Almalki is a PhD candidate in Health and Biomedical Informatics Centre (HaBIc), The University of Melbourne, and a lecturer in Jazan University, Saudi Arabia.

Dr Kathleen Gray is a senior researcher in the Health and Biomedical Informatics Centre (HaBIc), The University of Melbourne.

Professor Fernando Martin-Sanchez is the director of the Health and Biomedical Informatics Centre (HaBIc), The University of Melbourne.

## Declaration

The publication costs for the publication of this paper has been funded by the Health and Biomedical Informatics Centre (HaBIc), The University of Melbourne.

This article has been published as part of *Health Information Science and Systems *Volume 3 Supplement 1, 2015: Proceedings of the Health Informatics Society of Australia Big Data Conference (HISA 2013). The full contents of the supplement are available online at http://www.hissjournal.com/supplements/3/S1/
